# Patient perceptions of postoperative scarring after gender-affirming mastectomy: A cross-sectional study

**DOI:** 10.1016/j.jpra.2026.05.049

**Published:** 2026-06-06

**Authors:** Lindsay A. Tao, Carolyn B. Cafro, Esther A. Kim

**Affiliations:** a Department of Surgery, Division of Plastic Surgery. San Francisco, University of California, San Francisco, CA, USA; b University of Rochester School of Medicine and Dentistry. Rochester, NY, USA

**Keywords:** Gender-affirming mastectomy, Transgender surgery, Scar orientation. incision pattern, Aesthetic outcomes, Patient preferences, Top surgery

## Abstract

**Purpose:**

Double-incision gender-affirming mastectomies (GAMs) can produce prominent chest scars. While scarring may affect body image, confidence, and social participation, patient perceptions remain incompletely characterized. This study aimed to evaluate patient perceptions and psychosocial experiences related to post-GAM scarring.

**Methods:**

A cross-sectional survey was distributed to patients who underwent GAM. Participants rated statements regarding embarrassment, dissatisfaction, stigmatization, and social impact of scars using a five-point Likert scale. Additional items assessed scar width, thickness, color, and directionality. Free text responses were analyzed using thematic analysis. Responses were summarized descriptively, and Likert-scale data were dichotomized for exploratory chi-squared analysis.

**Results:**

Ninety-six responses were analyzed. Most participants were in their twenties (43%) or thirties (28%), identified as transgender men (66%) or non-binary (32%), and underwent double-incision mastectomy with free nipple graft (90%). The majority were White (67%), followed by Latinx (10%) and Asian (9%). Most participants were receiving hormone therapy (88%), and hypertrophic scarring was reported in 19%. Overall perceptions were generally favorable, with 86% denying embarrassment and 81% denying dislike of their scars (both p < 0.001). Although more participants preferred thinner scars, this difference was not statistically significant (p = 0.063). Concerns most often related to scar thickness, color, and length. Approximately 29% of participants reported stigmatization related to their scars, and 28% reported limiting shirtless activities because of scar visibility (both p < 0.001). Thematic analysis identified five primary domains: 1) scars as a source of pride and affirmation; 2) alignment between physical appearance and gender identity; 3) external perception and stigma; 4) acceptance of scars as inherent to surgery; and 5) dissatisfaction due to scar quality.

**Conclusion:**

Most patients reported neutral or positive perceptions of postoperative scarring following GAM. However, a subset of participants reported dissatisfaction related to scar quality and external perception. These findings suggest that postoperative scarring is experienced variably and may be influenced by both scar quality and social context.

## Introduction

Gender-affirming surgery is an important component of healthcare for transgender and gender diverse (TGD) individuals, with chest masculinization surgery, or gender-affirming mastectomy (GAM), among the most commonly performed procedures.^1^^,^^2^ Prior studies have demonstrated high postoperative satisfaction rates and improvements in gender dysphoria, body image, and quality of life following GAM.^3^^,^^4^ Despite these favorable outcomes, many GAM techniques, particularly double-incision mastectomy with free nipple grafting, result in prominent and permanent chest scars.^5^ While these scars represent an expected component of surgery, their long-term psychosocial impact and patient interpretation remain incompletely characterized.

In broader surgical and dermatologic literature, visible scarring has often been associated with dissatisfaction, self-consciousness, and reduced quality of life.^6^, ^7^, ^8^, ^9^ However, scar perception is highly context dependent and may be shaped by both physical scar characteristics and social experience. TGD individuals represent a particularly relevant population in which to examine these outcomes, as visible postoperative scars may intersect with body image, gender expression, and concerns regarding stigma or unwanted disclosure of transgender identity.^10^, ^11^, ^12^, ^13^ At the same time, scars may also be viewed positively as markers of gender affirmation or personal transition.^13^

Despite increasing utilization of gender-affirming surgery, limited literature has directly evaluated patient-reported perceptions of postoperative scarring following GAM. Prior work has explored scar-related experiences through online forums and social media analyses, but direct patient-reported assessment remains limited.^13^ Improved understanding of how patients perceive postoperative scars may help inform preoperative counseling, scar management strategies, and patient-centered postoperative care. The purpose of this study was to evaluate patient perceptions of postoperative scarring following GAM, with particular focus on psychosocial impact, satisfaction, and perceived stigma. We additionally sought to identify scar characteristics associated with dissatisfaction and to contextualize these findings through thematic analysis of patient-reported experiences.

## Methods

### Study design

This cross-sectional survey study was approved by the Institutional Review Board at the University of California, San Francisco (IRB #14–14,439). Eligible participants were adults identifying as transgender men or nonbinary who had previously undergone GAM. Participants were current or former patients of the senior author, identified through clinical records, and contacted via institutional patient email lists. Implied consent was obtained via participants’ decision to proceed with the survey. Participation was voluntary and uncompensated. Given the exploratory nature of this study and the absence of existing validated instruments specifically evaluating psychosocial perceptions of postoperative scarring following GAM, a study-specific questionnaire was developed to assess patient-reported scar perceptions and experiences.

### Survey instrument

The survey was developed by the study team based on clinical experience and prior observations of patient-reported concerns related to postoperative scarring. Because existing scar assessment tools primarily evaluate scar characteristics and associated symptoms, a study-specific questionnaire was designed to better characterize patients’ subjective perceptions of their scars.^14^

Item development was informed by previously established dermatologic patient-reported outcome measures, including the Dermatology Life Quality Index and Skindex-16, with adaptation of domains relevant to scar satisfaction, embarrassment, perceived stigma, and impact on daily and social activities.^8^^,^^9^ Responses were recorded using a five-point Likert scale ranging from strongly disagree to strongly agree. Additional items assessed specific scar characteristics: perceived width, thickness, contour, color, and directionality. Participants were able to submit free-text responses describing their experiences.

Demographic and surgical data were collected, including age, gender identity, race/ethnicity, mastectomy type, time from surgery, hormone therapy use, and hormone therapy duration. Descriptive data regarding postoperative complications were also collected, including hematoma, seroma, surgical site infection, hypertrophic scarring, and nipple graft loss. Postoperative interventions including Kenalog treatment and revision procedures were additionally recorded. However, these variables were not analyzed in relation to scar perception outcomes or satisfaction measures given the exploratory design and modest sample size. The survey was administered anonymously via Qualtrics (Provo, UT, USA), and all responses were stored securely in de-identified form.

### Data collection

A total of 436 eligible patients were invited to participate via email. Of these, 113 individuals initiated the survey, and 96 completed all questions, resulting in a response rate of 22%. Responses were collected over a six-week period, and incomplete surveys were excluded from analysis. Given the modest response rate and single-center design, findings should be interpreted with consideration for potential selection bias and limited generalizability.

### Statistical analysis

Descriptive statistics were used to summarize participant demographics and survey responses. Likert-scale responses were collapsed into agreement (strongly agree/agree) and disagreement (strongly disagree/disagree), with neutral responses excluded from inferential analyses. Given the exploratory nature of the study and modest sample size, dichotomization was performed to facilitate descriptive interpretation of overall response patterns. For each survey item, chi-squared goodness-of-fit testing was used to evaluate whether the observed distribution of agreement versus disagreement differed from an expected equal distribution. These analyses were intended to provide exploratory descriptive comparisons rather than establish causal or predictive relationships. Statistical significance was defined as p < 0.05. All analyses were performed using Microsoft Excel (Microsoft Corp., Redmond, WA) and R version 4.1.1 (R Foundation for Statistical Computing, Vienna, Austria).

Qualitative free-text responses were analyzed using reflexive thematic analysis.^15^ Two reviewers independently performed open coding to identify recurrent themes, later refined through iterative consensus. Given the exploratory qualitative design, thematic saturation was not formally assessed. Consistent patterns were observed across responses.

## Results

### Participant characteristics

Of 436 eligible individuals, a total of 96 participants completed the survey and were included in the final analysis. Most respondents were between 20 and 29 years (43%) or 30–39 years (28%). The majority identified as transgender men (66%), followed by non-binary (30%), then other (4%). All participants were assigned female at birth. All participants received GAM, with double-incision mastectomy with free nipple grafting (90%) as the most common technique. The remaining procedures included double-incision mastectomy without free nipple grafting (7%) and periareolar mastectomy (3%). The mean time from surgery was 4 years (range 1 month-11 years). Most participants were more than two years postoperative (63%), with smaller proportions within 6 months (21%), 6–12 months (10%), and 1–2 years (7%). Because a subset of participants were within 6 months of surgery, some responses may reflect perceptions prior to full scar maturation. The cohort was predominantly White (67%), followed by Latinx (10%) and Asian (9%) individuals. Most participants were receiving hormone therapy at the time of survey completion (88%), with mean hormone therapy duration of 26 ± 25 months. Postoperative complications included hypertrophic scarring (19%), partial nipple graft loss (16%), hematoma (9%), seroma (5%), surgical site infection (4%), and complete nipple graft loss (3%). Postoperative interventions included Kenalog treatment in 16% of participants and revision procedures in 11%, including nipple revision, scar revision, dog ear excision, and other minor in-clinic revisions (Table 1).

**Table 1:** Patient demographics.

Demographic	N (%)
All Participants	96 (100)
*Age (yr)*	
18–19	6 (6.25)
20–29	41 (42.71)
30–39	27 (28.13)
40–49	11 (11.46)
50+	11 (11.46)
*Gender*	
Transgender Man	63 (65.62)
Non-binary	29 (30.21)
Other	4 (4.17)
*Incisional Method of GAM*	
Double Incision w/ FNG	86 (89.58)
Double Incision w/out FNG	7 (7.29)
Periareolar	3 (3.13)
*Ethnicity*	
White	64 (66.67)
Latinx	10 (10.42)
Asian	9 (9.38)
Mixed	10 (10.42)
Black	1 (1.04)
Middle Eastern	1 (1.04)
Other	1 (1.04)
*Time from surgery*	
<6 months	20 (20.83)
6–12 months	10 (10.42)
1–2 years	7 (7.29)
2+ years	59 (63.44)
*Postoperative Complications*	
Hematoma	9 (9.38)
Seroma	5 (5.21)
Surgical Site Infection	4 (4.17)
Hypertrophic Scarring	18 (18.75)
Partial nipple graft loss	14 (16.28)
Complete nipple graft loss	3 (3.49)
*Postoperative Interventions*	
Kenalog Treatment	15 (15.62)
Revision Procedure	11 (11.46)
*Hormone Therapy*	
Receiving hormone therapy	84 (87.50)
Hormone therapy duration, mean ± SD (months)	26 ± 25.14

GAM: Gender-affirming mastectomy; FNG: Free nipple graft.

### Overall scar perceptions

**Fig. 1:**
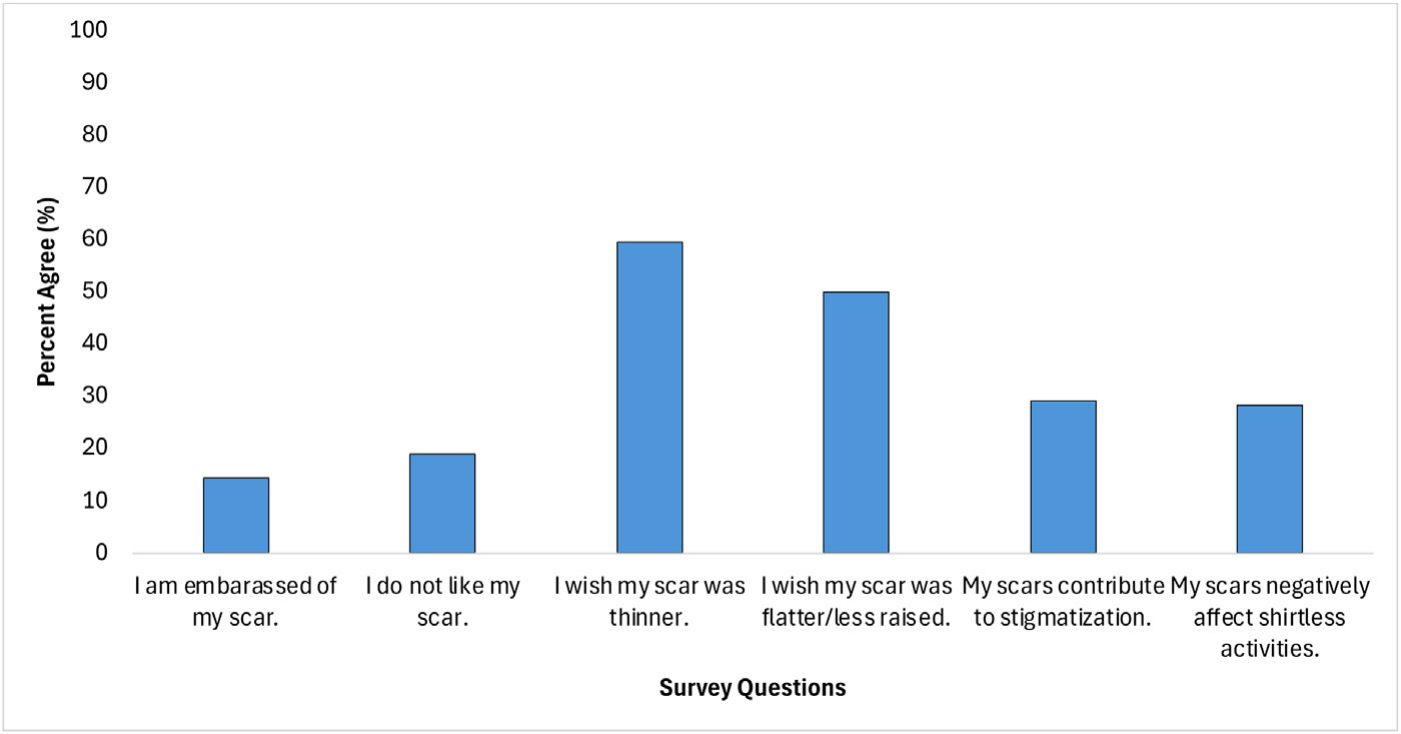
**Percentage agreement with survey questions regarding postoperative scar perception.** Agreement defined as the sum of "Strongly Agree" and "Agree" responses. Disagreement defined as the sum of "Strongly Disagree" and "Disagree" responses. Neutral responses excluded from chi-squared analysis. Preferences for thinner scars (p = 0.091) and preferences for flatter scars (p = 1) not statistically significant. All other comparisons p < 0.001.

Overall perceptions of postoperative scars were favorable, with low rates of agreement with negative perception items (Fig. 1). The majority of participants denied feeling embarrassed by their scars (86%, p < 0.001) and denied disliking their scars (81%, p < 0.001) (Table 2). These findings indicate that negative emotional responses to scarring were relatively uncommon within this cohort.

**Table 2:** Participant Response Summary.

Survey Question	Agreement	Disagreement	*p*-value
I am embarrassed of my scar.	12 (14)	71 (86)	**<0.001**
I do not like my scar.	15 (19)	64 (81)	**<0.001**
I wish my scar was thinner.	47 (59)	32 (41)	0.091
I wish my scar was flatter/less raised.	35 (50)	35 (50)	1
My scars contribute to stigmatization.	23 (29)	56 (71)	**<0.001**
My scars negatively affect shirtless activities.	23 (28)	58 (72)	**<0.001**

Data presented as n (%). Agreement defined as the sum of "Strongly Agree" and "Agree" responses. Disagreement defined as the sum of "Strongly Disagree" and "Disagree" responses. Neutral responses excluded from chi-squared analysis.

**Fig. 2:**
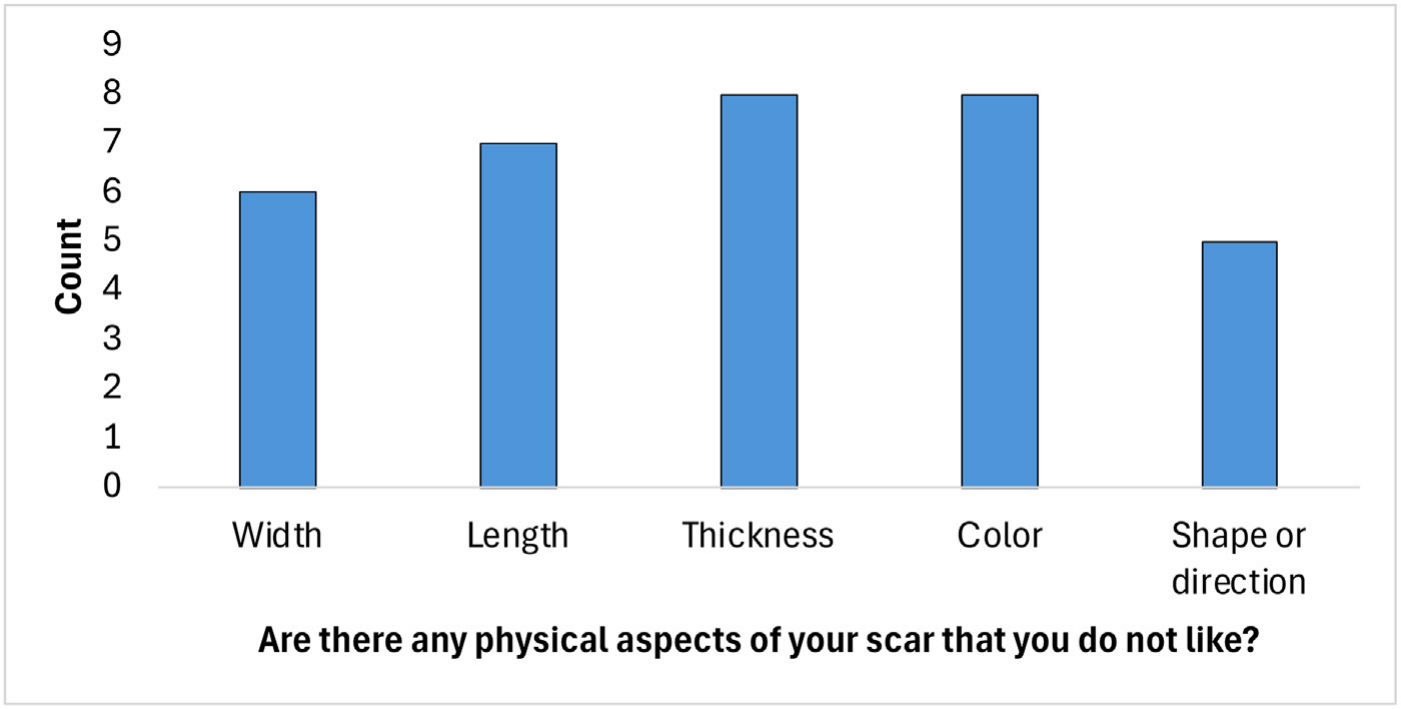
**Count of physical aspects disliked in scars.** Total count of participant responses who chose to indicate specific scar characteristics they disliked.

Despite generally favorable perceptions, participants expressed preferences for improvement in specific scar characteristics. A greater proportion of participants endorsed a desire for thinner scars (59%vs 41%), although this difference did not reach statistical significance (p = 0.091). Preferences regarding scar flatness were evenly distributed (50%vs 50%, p = 1.0) (Table 2). Participants most frequently identified scar thickness, color, and length as areas of concern, with additional concerns related to width and directionality (Fig. 2).

A subset of participants reported negative psychosocial effects. Approximately 29% of participants reported that their scars contributed to feelings of stigmatization, and 28% reported limiting participation in shirtless activities due to their scars (both p < 0.001) (Table 2). These findings suggest variability in how scars were perceived socially and functionally, despite overall favorable perceptions reported by most participants.Total participant response counts are Ysummarized in Supplementary Table 1 and illustrated in Supplementary Figures 1 and 2.

### Subgroup analysis by gender identity

Subgroup analysis comparing transgender men and nonbinary participants demonstrated broadly similar overall patterns of scar perception across groups. Rates of embarrassment (18%vs 7%), dislike of scars (26%vs 4%), perceived stigma (35%vs 19%), and limitation of shirtless activities (30%vs 26%) were numerically higher among transgender men compared to nonbinary participants. Preferences for scar characteristics, including desire for thinner (61%vs 56%) or flatter scars (52%vs 45%), were comparable between groups. Given the limited subgroup sample sizes, these findings should be interpreted descriptively, and no statistically significant differences were identified between groups (Table 3).

**Table 3:** Participant response summary by gender identity.

	Transgender male (n = 62)		Nonbinary (n = 29)	
	Agreement	Disagreement	Agreement	Disagreement
I am embarrassed of my scar.	10 (18)	45 (82)	2 (7)	26 (93)
I do not like my scar.	14 (26)	39 (74)	1 (4)	25 (96)
I wish my scar was thinner.	33 (61)	21 (39)	14 (56)	11 (44)
I wish my scar was flatter/less raised.	25 (52)	23 (48)	10 (45)	12 (55)
My scars contribute to stigmatization.	18 (35)	34 (65)	5 (19)	22 (81)
My scars negatively affect shirtless activities.	16 (30)	38 (70)	7 (26)	20 (74)

Data presented as n (%). Agreement defined as the sum of "Strongly Agree" and "Agree" responses. Disagreement defined as the sum of "Strongly Disagree" and "Disagree" responses. Percentages calculated among respondents excluding neutral responses.

### Thematic analysis

Free-text responses were provided by all 96 participants and were included in thematic analysis. Five primary themes were identified, with representative quotes highlighted:

1. *Scars as a source of pride and affirmation.* Participants described scars as positive markers of identity, transition, and personal achievement. Scars were frequently framed as meaningful markers of one’s transition and living authentically. Some participants also expressed positive aesthetic perceptions, describing their scars as visually appealing or “cool”. One participant noted, “My scars are a point of pride for me. They're a reminder that I fought hard to live as myself and no one can take them from me.” Another shared, “I feel pride in my scars and see them as a mark in my journey towards who I am.”
2. *Alignment between physical appearance and gender identity.* Participants frequently described postoperative changes as facilitating a greater sense of alignment between their physical appearance and gender identity. This was often expressed as a newfound sense of comfort or completeness within their body. One participant shared, “This procedure has allowed me to feel fully in my body for the first time in nearly 30 years.” Others described improved satisfaction despite residual self-consciousness: “I don't feel embarrassed by my surgery scar. While I'm sometimes self-conscious being topless in public, I'm more satisfied with my chest now than I was pre-surgery.”
3. *External perception and stigma.* Participants relayed concerns related to how their scars might be perceived by others. Some participants described visible scarring as a marker of transgender identity, with implications for safety, discrimination, and discomfort in public settings. One participant noted, “I don't find them inherently shameful, but they are a very clear indicator that I'm transgender, and that can put me at risk. The scar itself I don't really mind. Almost all of my concerns about it are to do with perception from other people.” This was often accompanied by behavioral adaptations, with another participant stating, “I'm not embarrassed, but I stay covered around people I don't know to avoid discrimination.”
4. *Acceptance of scars as inherent to surgery.* Many participants described their scars in neutral terms, emphasizing them as an expected outcome of the procedure rather than a source of distress. Scars were understood to be an unavoidable tradeoff for achieving desired bodily concordance. One participant stated, “I got surgery knowing there would be a scar. So I expected a scar and I was fine with it.” Similarly, another shared, “I would rather not have a scar, but I knew I would have one when I got the surgery and am accepting it.”
5. *Dissatisfaction related to scar quality.* A subset of participants expressed dissatisfaction related to specific scar characteristics, most commonly thickness, color, and contour irregularities such as hypertrophic scarring or dog ears. These concerns were tied to healing outcomes rather than the surgical decision itself. One participant noted, “I am happy I have had the surgery but my scars are very raised.” Others described concerns related to healing: “The shape doesn’t bother me but my scars stretched and they’re very red on pale skin so I have large scars that are extremely noticeable.”

## Discussion

This study suggests that patient perceptions of postoperative scarring following GAM were generally favorable, with most participants denying embarrassment or dissatisfaction with their scars. However, a subset reported ongoing psychosocial impact, including experiences of stigmatization and limitations in activities where scars are visible. Across both quantitative and qualitative findings, negative internal perceptions of scars appeared less common than concerns related to scar quality and external perception. Participants variably described scars as either affirming markers of identity or as potential sources of vulnerability depending on context. Most participants were over two years postoperative, suggesting that these findings may reflect longer-term perceptions following scar maturation. Taken together, these findings suggest that postoperative scar perception may be influenced less by the presence of scars alone and more by scar quality and the social contexts in which scars are encountered.

In contrast to conventional surgical and dermatologic paradigms, which often frame scarring as a negative outcome associated with dissatisfaction and self-consciousness, many participants in this study described their scars in neutral or positive terms.^6^^,^^16^^,^^17^ Quantitatively, rates of embarrassment and dislike were low, and thematic analysis further suggested that scars were frequently interpreted as markers of identity and transition. These findings are consistent with prior work demonstrating that gender-affirming surgical interventions are associated with high levels of patient satisfaction and psychosocial benefit.^3^^,^^18^^,^^19^ Prior studies have shown that postoperative complications do not significantly diminish patient-reported outcomes following chest masculinization surgery, suggesting that overall satisfaction may reflect broader alignment with gender identity rather than isolated aesthetic concerns.^20^ Similarly, patient-reported outcome studies in scar-related quality of life demonstrate that the impact of visible scarring is heterogeneous and context dependent rather than uniformly negative.^21^

Our findings extend prior literature by suggesting that postoperative scars may, for some patients, become incorporated into the broader process of gender affirmation. While prior qualitative work has highlighted that scarring may contribute to dysphoria, visibility concerns, or self-consciousness,^13^ many participants in our cohort instead described scars as affirming or personally meaningful. These findings suggest that postoperative scarring following GAM may be experienced differently from scarring in other surgical settings, as scars exist within a broader context of gender affirmation and identity alignment.

Dissatisfaction, when present, was most closely related to specific features of scar appearance rather than the presence of scars themselves. Participants most frequently expressed preferences for thinner scars, and qualitative responses highlighted concerns related to hypertrophic scarring, widening, and pigmentation. These findings suggest that satisfaction may be influenced less by scar visibility alone and more by how scars heal and integrate with surrounding anatomy. Hypertrophic scarring represents a common aberration of wound healing characterized by excessive collagen deposition and raised, symptomatic scars.^22^ Emerging evidence suggests that both surgical technique and patient-specific factors may influence the risk of hypertrophic scarring following GAM, emphasizing the importance of individualized perioperative management.^23^ Current management strategies for hypertrophic and cosmetically unfavorable scars include silicone-based therapies, corticosteroid injections, and laser treatments, often employed in a multimodal fashion to optimize outcomes.^24^^,^^25^ Emerging evidence suggests that androgen exposure may influence wound healing and scar formation through effects on inflammatory signaling, extracellular matrix remodeling, fibroblast activity, and collagen deposition.^7^^,^^26^ Recent studies in transmasculine patients undergoing GAM have additionally suggested possible associations between perioperative testosterone exposure and hypertrophic scar formation, although findings remain mixed and prospective clinical evidence remains limited.^26^ Most participants in the present cohort were receiving hormone therapy at the time of survey completion; however, associations between hormone therapy variables and scar perception outcomes were not evaluated in the present study. Together, these findings highlight scar quality as a potentially modifiable contributor to postoperative satisfaction.

Salient concerns centered on external perceptions of post-GAM scarring. Thematic analysis suggested that some participants viewed scars as visible indicators of transgender identity, raising concerns regarding unwanted disclosure, discrimination, or discomfort in public settings. Participants often described modifying behavior, such as avoiding shirtless environments, not necessarily because of dissatisfaction with their bodies, but because of concern regarding how scars may be perceived by others. This distinction highlights the difference between internal acceptance of scars and external social vulnerability. Prior literature has similarly demonstrated that visible scarring following gender-affirming procedures may affect an individual’s ability to “blend” within their affirmed gender and contribute to psychosocial distress or safety concerns.^13^^,^^27^ More broadly, transgender individuals face disproportionately high rates of stigma and discrimination, which may further shape perceptions of visible surgical markers.^10^^,^^11^ Efforts to improve cultural understanding and acceptance of visible differences may help improve the broader social environment in which these scars are encountered.

Patterns of scar perception were broadly similar between transgender men and nonbinary participants, suggesting relative consistency of findings across gender identities within this cohort. Although transgender men reported numerically higher rates of negative perceptions, these differences were not statistically significant and should be interpreted cautiously given the limited subgroup sample size. Prior studies suggest that while both transgender and nonbinary individuals report high levels of satisfaction following chest surgery, nonbinary patients may have more heterogeneous goals and experiences.^28^, ^29^, ^30^ This variability may influence how postoperative scars are perceived and incorporated into one’s sense of embodiment or identity.

This study supports a nuanced approach to perioperative counseling and postoperative care in GAM. Rather than focusing solely on the inevitability of visible scarring, clinicians may benefit from discussing scars within the broader context of patient goals, expectations, and lived experience. Preoperative counseling may also include discussion of how individuals anticipate relating to scars over time, including both aesthetic and psychosocial considerations. From a surgical standpoint, these findings reinforce the importance of scar optimization, where attention to incision planning, tension management, and longitudinal scar care may meaningfully influence patient satisfaction. Finally, these findings suggest that the patient experience extends beyond the operative result itself, and may also be shaped by broader social perceptions and experiences related to visible scarring.

Several limitations should be considered. The cross-sectional design limits assessment of how perceptions of scarring evolve over time, as experiences may change with scar maturation and longer-term psychosocial adaptation. Additionally, 21% of participants were within 6 months of surgery, and therefore some responses may reflect perceptions prior to full scar maturation. The modest response rate and single-center recruitment design introduce potential selection bias and may limit generalizability. The cohort was also predominantly White, which may limit applicability to populations with differing risks of hypertrophic or keloid scarring. Although informed by existing dermatologic QOL instruments, this study-specific survey was not formally validated. Likewise, dichotomization of Likert-scale responses reduced response granularity and may have limited sensitivity to more nuanced perceptions. Although descriptive data regarding hormone therapy use, postoperative complications, and postoperative interventions were collected, the present study was not powered to evaluate associations between these variables and scar perception outcomes. Additionally, detailed revision-specific analyses were not performed despite their potential influence on postoperative satisfaction and scar perception. Additionally, scar perception is influenced by individual variability in wound healing and scar biology, including predisposition to hypertrophic scarring. While thematic analysis provided important insight into patient experiences, qualitative findings remain inherently interpretive and thematic saturation was not formally assessed. Finally, perceptions of scarring are likely shaped not only by surgical outcomes, but also by broader social and geographic context. As this cohort was drawn from a single academic center, these findings may not fully reflect the experiences of individuals in less affirming or resource-rich environments.

## Conclusion

Most patients undergoing GAM reported neutral or positive perceptions of postoperative scarring. However, a subset of participants reported dissatisfaction related to scar quality and external perception. These findings suggest that postoperative scarring is not uniformly experienced as detrimental but rather represents a complex and individualized component of surgical outcomes following GAM. Efforts aimed at optimizing scar quality and supporting patients in navigating the broader social context of visible scarring may further improve patient-centered postoperative outcomes.

## Source of funding

None.

## Ethical approval

This study was approved by the University of California, San Francisco Institutional Review Board (14-14439) and conforms to the World Medical Association Declaration of Helsinki.

## CRediT authorship contribution statement

**Lindsay A. Tao:** Conceptualization, Investigation, Methodology, Project administration, Data curation, Formal analysis, Writing – original draft, Visualization, Writing – review & editing. **Carolyn B. Cafro:** Conceptualization, Data curation, Formal analysis, Investigation, Methodology, Project administration, Resources, Visualization, Writing – original draft, Writing – review & editing. **Esther A. Kim:** Conceptualization, Supervision, Methodology, Writing – review & editing, Project administration.

## Declaration of competing interest

None.

## Appendix Supplementary materials

Image, application 1
