# Supplementary material for: Patient perceptions of postoperative scarring after gender-affirming mastectomy: A cross-sectional study
Source: JPRAS Open. 2026 Jun 6;51:118–28. doi: 10.1016/j.jpra.2026.05.049 (PMC13320309; doi:10.1016/j.jpra.2026.05.049)

**Supplementary Figures and Tables**

**Supplementary Table 1. Participant Response Count**

| **Survey Question** | **Strongly Agree** | **Agree** | **Neutral** | **Disagree** | **Strongly Disagree** |
| --- | --- | --- | --- | --- | --- |
| I am embarrassed of my scar. | 5 | 7 | 13 | 27 | 44 |
| I do not like my scar. | 8 | 7 | 17 | 21 | 43 |
| I wish my scar was thinner. | 17 | 30 | 17 | 17 | 15 |
| I wish my scar was flatter/less raised. | 14 | 21 | 26 | 19 | 16 |
| My scars contribute to stigmatization. | 8 | 15 | 17 | 22 | 34 |
| My scars negatively affect shirtless activities. | 8 | 15 | 15 | 24 | 34 |

**Supplementary Figure 1. Heatmap of participant response percents.**

**Supplementary Figure 2. Response percents for survey questions.**


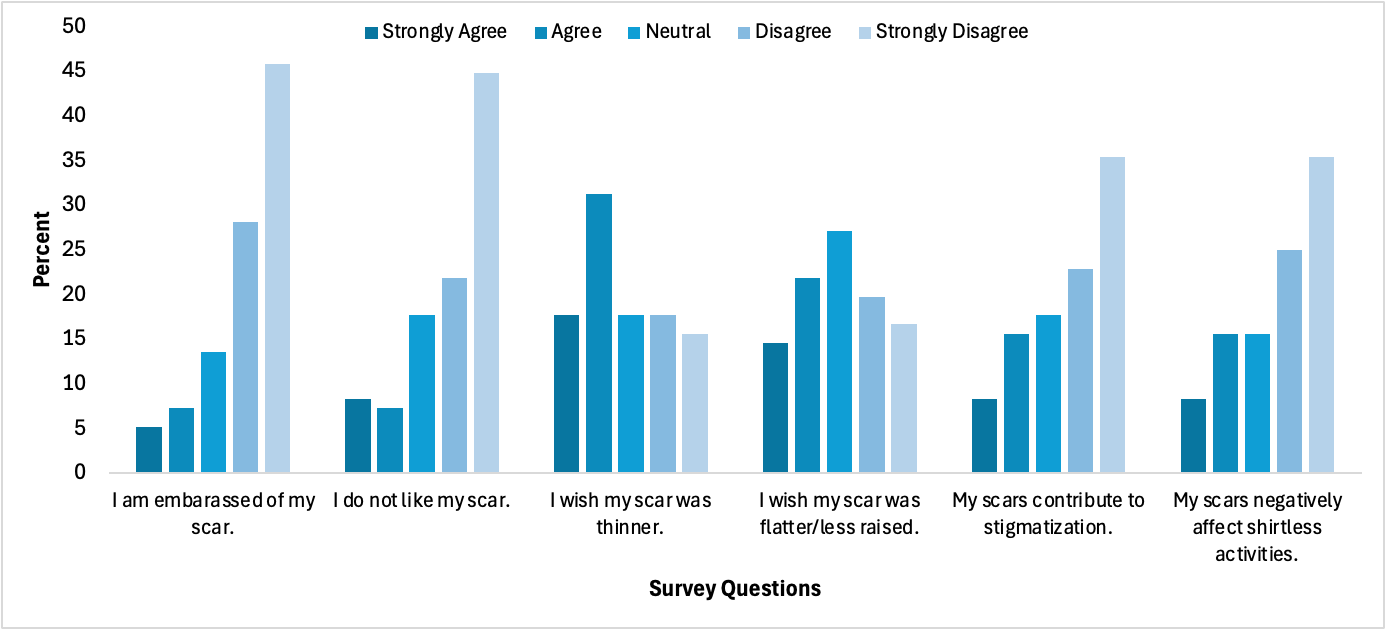

Supplement: Supplementary file 1 [file mmc1.docx]
